# Autophagy is a signature of a signaling network that maintains hematopoietic stem cells

**DOI:** 10.1371/journal.pone.0177054

**Published:** 2017-05-09

**Authors:** Michelle Nguyen-McCarty, Peter S. Klein

**Affiliations:** 1 Cell and Molecular Biology Graduate Group, University of Pennsylvania Perelman School of Medicine, Philadelphia, Pennsylvania, United States of America; 2 Department of Medicine (Hematology/Oncology), University of Pennsylvania Perelman School of Medicine, Philadelphia, Pennsylvania, United States of America; Emory University, UNITED STATES

## Abstract

Hematopoietic stem cells (HSCs) are able to self-renew and to differentiate into all blood cells. HSCs reside in a low-perfusion niche and depend on local signals to survive and to maintain the capacity for self-renewal. HSCs removed from the niche are unable to survive without addition of hematopoietic cytokines and rapidly lose their ability to self-renew. We reported previously that inhibition of both GSK-3 and mTORC1 is essential to maintain long-term HSCs *ex vivo*. Although Wnt/β-catenin signaling downstream of GSK-3 is required for this response, the downstream effectors of mTORC1 remain undefined. We now report that HSCs express a pro-autophagic gene signature and accumulate LC3 puncta only when both mTORC1 and GSK-3 are inhibited, identifying autophagy as a signature for a signaling network that maintains HSCs *ex vivo*. In addition, these conditions sustain HSC repopulating function despite an increased rate of global translation. Together, these findings provide new insight into the relative contributions of various mTORC1 outputs toward the maintenance of HSC function and build upon the growing body of literature implicating autophagy and tightly controlled protein synthesis as important modulators of diverse stem cell populations.

## Introduction

Stem cells are defined by the ability to both self-renew and generate differentiated cells, enabling them to regenerate tissues under steady-state conditions and in response to injury. This essential function in lifelong tissue maintenance endows stem cells with exceptional potential for clinical applications. HSCs, for example, are responsible for maintaining the blood, and are used for the treatment of both congenital and acquired hematological disorders [1]. While tremendous progress has been made toward optimizing therapeutic use of HSCs, this is a rare population. Efforts to expand HSCs *ex vivo*, for example for umbilical cord transplants or for therapeutic genome editing, have been hampered by an incomplete understanding of the signaling networks that regulate HSC fate decisions and subsequent difficulty defining conditions that maintain HSC function beyond the niche [2].

HSCs reside in a low-perfusion niche [3–5], underscoring nutrient-sensing as an essential function. The evolutionarily conserved nutrient sensor mTORC1 antagonizes HSC function, as interventions that activate mTORC1, including loss of the negative regulators *Pten* [6], *Gsk3* [7], and *Tsc1* [8], lead to HSC proliferation followed by exhaustion and in some cases leukemogenesis [6, 9]. Developing a thorough understanding of mediators of mTORC1 signaling in this context will therefore be a critical step toward expansion of functional HSCs *ex vivo*.

mTORC1 regulates cell growth and proliferation through multiple effectors. The relative importance of each of these effectors to HSC function and fate decisions remains unclear. The best-characterized output of mTORC1 activation is the regulation of translation via activation of S6 kinase (S6K) [10] and inhibition of eIF4E binding protein (4E-BP) [11]. HSCs require a precise level of translation: they exhibit a low basal rate of translation compared to more differentiated hematopoietic cell populations, and either increasing or decreasing this rate impairs their engraftment upon transplantation [12, 13]. mTORC1 additionally promotes mitochondrial biogenesis through both translation-dependent [14] and -independent [15, 16] mechanisms. HSCs have been reported to have lower mitochondrial mass and membrane potential than other hematopoietic populations, with increases in these features correlating with loss of self-renewal capacity [17].

mTORC1 also suppresses autophagy, which is both a pro-survival mechanism in the context of nutrient starvation and an important quality control system in the turnover of old or damaged proteins and organelles. Autophagy is therefore a critical component of stem cell maintenance, differentiation, and aging [18], and disruption of this pathway has been linked to loss of function of a variety of stem cell populations, including embryonic, epidermal [19], skeletal muscle [20, 21], neural [22, 23], and hematopoietic [24, 25] stem cells. In the hematopoietic system, loss of the essential autophagy genes *Atg7* or *Atg5* impairs HSC function while promoting proliferation of hematopoietic progenitor cells, leading to bone marrow failure [24, 26]. Moreover, highlighting the essential role of nutrient-sensing in the reduced-perfusion HSC niche, HSCs activate autophagy to survive cytokine starvation, while progenitors fail to activate autophagy and instead undergo apoptosis [27]. These findings indicate a unique requirement for autophagy in the function of HSCs as opposed to other hematopoietic cell populations.

Despite the extensive body of literature characterizing these distinct outputs of mTORC1 signaling in HSCs, the role of each in HSC maintenance remains unclear. The complexity of the HSC niche and consequent challenge maintaining HSCs *ex vivo* have constrained efforts to address this question. Previous work from our laboratory showed that HSCs are maintained *ex vivo* in cytokine-free conditions when GSK-3 and mTORC1 are inhibited [28]. Inhibition of GSK-3 activates downstream Wnt/β-catenin signaling, and β-catenin is required for HSC maintenance in this setting, but the pathway(s) downstream of mTORC1 that contribute to this response have not been identified. We have investigated the complex signaling network downstream of mTORC1 associated with the maintenance of long-term HSCs. We find that activation of autophagy is uniquely associated with conditions that maintain self-renewing HSCs.

## Results

### Cell-autonomous regulation of HSC function by GSK-3 and mTORC1

**Fig 1:**
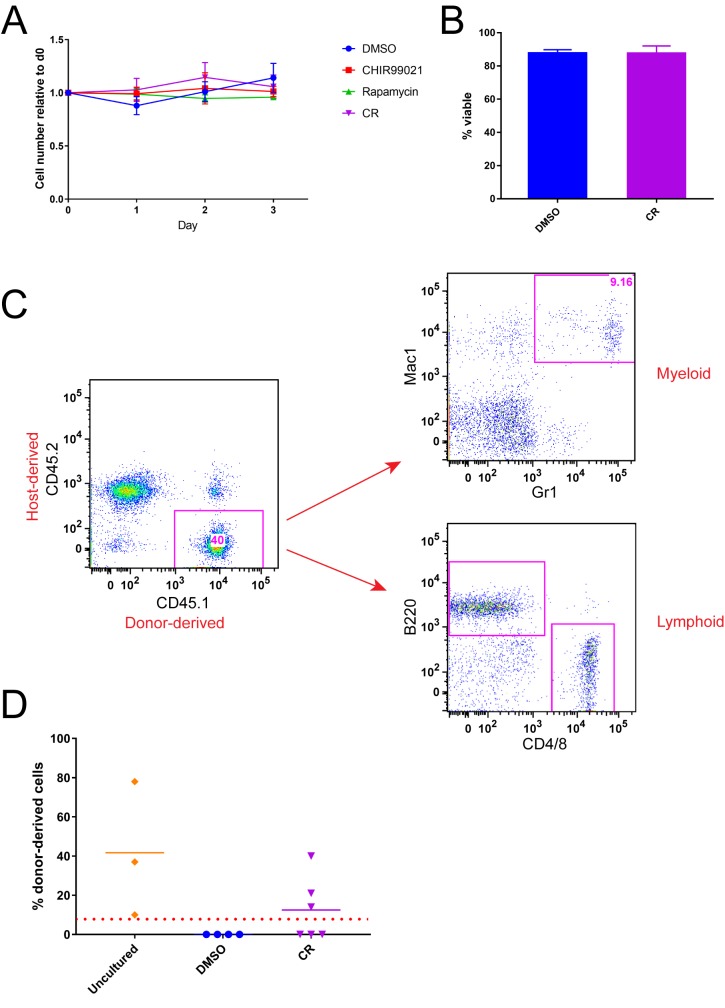
Cell-autonomous regulation of HSC function by GSK-3 and mTORC1. (A) HSCs were cultured for 3 d, and cell number relative to day 0 was determined visually. (B) Viability of HSCs cultured in control medium or CR after 7 d culture, determined by Trypan Blue exclusion. (C, D) HSCs were cultured for 7 d and then transplanted with competitor cells into lethally irradiated hosts. Peripheral blood was collected at 24 weeks post-transplant, and multilineage potential of donor-derived (CD45.1^+^) cells was determined by flow cytometry for lineage-specific markers as indicated (C). Flow cytometry data shown are from one recipient representative of CR-cultured HSCs. Long-term engraftment of freshly isolated HSCs or of HSCs cultured in vehicle or CR was determined by flow cytometry for donor-derived (CD45.1^+^) cells following competitive transplant (D). Chimerism data shown were collected 24 weeks after transplant. Each symbol represents the results from an individual mouse. Red dotted line indicates 5% threshold defining recipients as positive for donor-derived engraftment. Error bars represent S.E.M.

We previously reported that simultaneous GSK-3 and mTORC1 inhibition maintains HSC function *ex vivo* in hematopoietic stem and progenitor cells (HSPCs, c-Kit^+^ or Lin^-^Sca1^+^c-Kit^+^ [LSK]) [28]. While this fraction is enriched for HSCs, it is a heterogeneous population composed primarily of progenitor cells. To address a potential indirect effect of modulating GSK-3 and mTORC1, we sorted HSCs (LSK-CD48^-^CD150^+^ [LSK-SLAM]) and cultured them in serum-free, cytokine-free medium in the presence or absence of the GSK-3 inhibitor CHIR99021 and the mTORC1 inhibitor rapamycin (CR). Cell number did not significantly change during culture, and ~87% of cells remained viable after 7 d of culture (**Fig 1A and 1B**). This result is consistent with our previous observation that there is no increase in the fraction of apoptotic (Annexin V^+^) cells in control-treated cells compared to CR-treated cells [28]. To assess HSC function, we performed a competitive repopulation assay. CD45.1^+^ HSCs cultured in vehicle or CR for 7 d were injected with CD45.2^+^ competitor whole bone marrow cells into lethally irradiated recipients. HSCs cultured with CR retained both multilineage and long-term (up to 24 weeks) engraftment potential, while control cultured cells failed to engraft (**Fig 1C and 1D, S1 Fig**). (We have previously shown that neither CHIR99021 nor rapamycin alone maintains repopulating function in cultured HSPCs [Huang et al., 2012].) Importantly, we transplanted only 200 donor cells per mouse yet still observed engraftment, demonstrating the preservation of robust repopulating capacity in CR-cultured HSCs. These results indicate that GSK-3 and mTORC1 inhibition maintains stem cell function by acting directly on HSCs.

### mTORC1 activity and global translation during HSC maintenance

**Fig 2:**
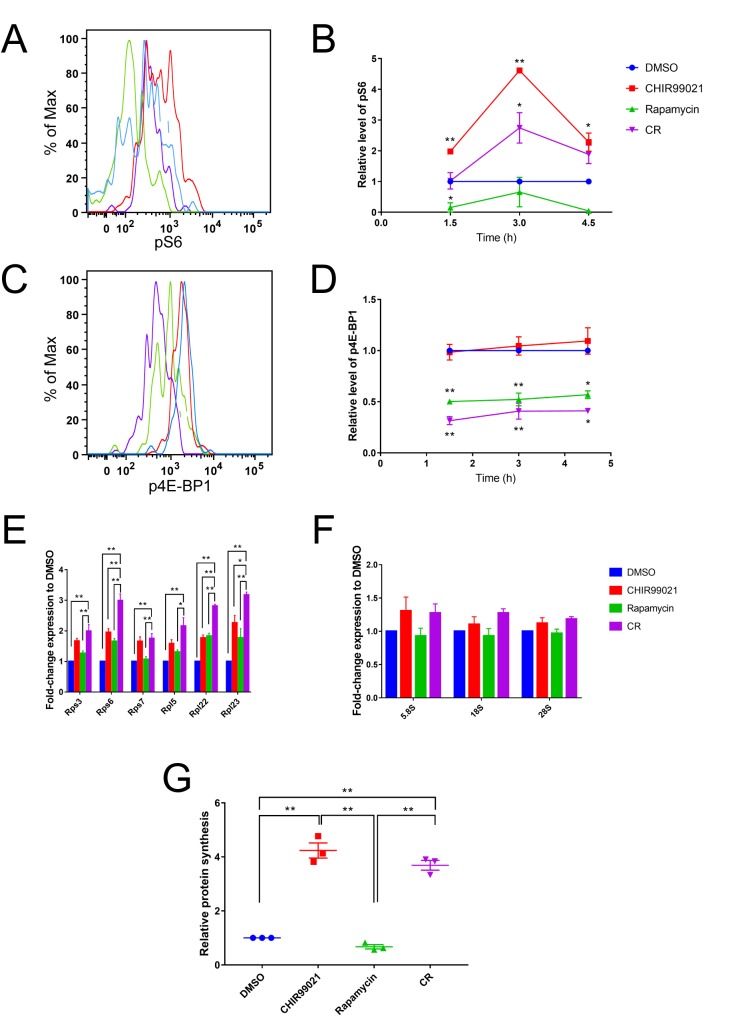
mTORC1 activity and global translation during HSC maintenance. (A-D) Representative flow cytometry histograms of pS6 (A) and p4E-BP1 (C) from 1.5 h time point, and their median fluorescence intensities relative to DMSO (B, D), measured by phospho-flow cytometry in the LSK-CD48^-^ fraction of lineage-depleted bone marrow cells at the indicated times after cells were isolated and placed into culture medium. Asterisks indicate statistical significance for comparison of treatment to DMSO within the same time point. (E, F), c-Kit^+^ cells were cultured for 24 h followed by RNA isolation for RT-PCR analysis. RT-PCR for ribosomal protein (E) and ribosomal RNA (F) gene expression relative to DMSO. (G) Lineage-depleted bone marrow cells were cultured for 24 h, and then rate of protein synthesis relative to DMSO was measured by incorporation of OP-Puro followed by flow cytometric analysis of the LSK-CD48^-^ fraction. Error bars represent S.E.M. Statistical significance for gene expression analyses was assessed using a one-way ANOVA followed by Dunnett’s test for multiple comparisons. Statistical significance for pS6 and p4E-BP1 median fluorescence intensities was assessed using a one-way ANOVA followed by Tukey’s test for multiple comparisons. * p < 0.05, ** p < 0.005.

mTORC1 regulates cell growth and metabolism through multiple independent effectors, the best-characterized of which is promotion of translation through activation of S6K and inhibition of 4E-BP. As HSCs require a precise level of translation [12, 13], we further explored the regulation of S6 and 4E-BP in cultured HSCs. The canonical readout for mTORC1 activity and regulation of translation is therefore measurement of phospho-S6 (pS6) and phospho-4E-BP1 (p4E-BP1) levels, which we assessed over time in LSK-CD48^-^ cells. (The CD150 epitope is destroyed during fixation and permeabilization [29].) GSK-3 suppresses mTORC1, and GSK-3 inhibition therefore activates mTORC1 [7, 28, 30]. Although we did not observe an effect on pS6 or p4E-BP1 using the inhibitors at our standard concentrations for HSC culture, CHIR99021 and rapamycin are typically used at three and 100 times higher concentrations, respectively. A correspondingly higher concentration of GSK-3 inhibitor increases phosphorylation of S6 in LSK-CD48^-^ cells, as shown by flow cytometric detection with a phospho-specific pS6 antibody (**Fig 2A and 2B**). Rapamycin inhibits S6 phosphorylation, as expected, and attenuates but does not completely inhibit increased S6 phosphorylation when combined with CHIR99021 (**Fig 2A and 2B**). Rapamycin also reduces 4E-BP1 phosphorylation in LSK-CD48^-^ cells. We moreover observe a trend toward a further decrease in p4E-BP1 in CR-treated cells, although the difference fails to reach statistical significance (**Fig 2C and 2D**). Simultaneous inhibition of GSK-3 and mTORC1 therefore achieves a level of mTORC1 activity that is distinct from that following inhibition of either alone.

mTORC1 regulates translation in part via S6K promotion of ribosomal gene transcription [31]. We therefore cultured c-Kit^+^ cells in vehicle, CHIR99021 (3 μM), rapamycin (5 nM), or both for 24 h and measured the expression of large and small ribosomal subunit protein genes by RT-PCR. CR-treated cells consistently express higher levels of all ribosomal protein genes assessed when compared to control or rapamycin-treated cells, and in most cases compared to CHIR99021-treated cells as well (**Fig 2E**). Importantly, ribosomal RNA expression is equal across conditions (**Fig 2F**). Together, these results demonstrate a specific enrichment for expression of ribosomal protein genes in CR-treated HSPCs, suggesting an elevated capacity for protein biosynthesis.

The increased expression of ribosomal protein genes in CR-cultured HSPCs was unexpected, as we predicted that rapamycin would block mTORC1-dependent activation of translation. However, the effective concentration of rapamycin in our culture system is lower than typically used. To address translation directly, we measured the rate of global protein synthesis by O-propargyl-puromycin (OP-Puro) incorporation into nascent polypeptides and fluorescent labeling [32] followed by flow cytometric analysis. We find a stepwise increase in the rate of translation as HSCs differentiate into more committed progenitor populations (**S2A Fig**), as previously reported [12]. We next measured OP-Puro incorporation in LSK-CD48^-^ cells after 3 h and 24 h culture with or without inhibitors. Bulk protein synthesis is elevated in CHIR99021-treated cells compared to cells cultured with DMSO or rapamycin alone, consistent with mTORC1 and S6K activation. However, protein synthesis was also increased in CR-treated cells (**Fig 2G, S2B Fig**). While this finding is consistent with the increased pS6 and increased ribosomal protein expression observed in CR, it is not consistent with suppression of global protein synthesis as a mechanism for CR maintenance of HSCs.

### Targeting S6K and eIF4E downstream of mTORC1 in HSC maintenance

**Fig 3:**
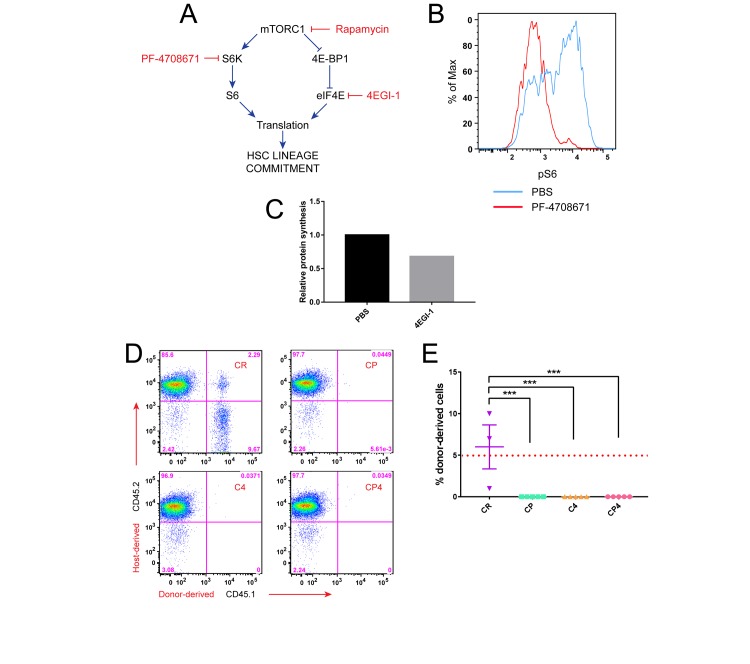
Targeting S6K and eIF4E downstream of mTORC1 in HSC maintenance. (A) Strategy to test inhibition of S6K or eIF4E in HSC culture: If the primary effect of rapamycin in HSC maintenance is to inhibit S6K and/or eIF4E, then direct inhibitors of these downstream effectors should be able to replace rapamycin and suppress lineage commitment. (B) Flow cytometric analysis of pS6 in the LSK-CD48^-^ fraction of lineage-depleted bone marrow cells treated with PF-4708671. (C) Rate of translation relative to vehicle in LSK-CD48^-^ cells treated with 4EGI-1, measured by OP-Puro incorporation. (D,E) LSK cells were cultured for 7 d and then transplanted with competitor cells into lethally irradiated hosts. Donor-derived engraftment was determined by flow cytometry for CD45.1^+^ cells in peripheral blood from recipients of cultured HSPCs following competitive transplant. Chimerism data shown were collected 8 weeks after transplant. Flow cytometry data shown are from one recipient representative of each transplant group (D). Each symbol represents the result from an individual mouse, and red dotted line indicates 5% threshold defining recipients as positive for donor-derived engraftment (E). CP, CHIR99021 + PF-4708671; C4, CHIR99021 + 4EGI-1; CP4, CHIR99021 + PF-4708671 + 4EGI-1. Error bars represent S.E.M. Statistical significance was assessed using a one-way ANOVA followed by Dunnett’s test for multiple comparisons. *** p < 0.0005.

While these findings argue against suppression of protein synthesis as a mechanism of HSC maintenance *ex vivo*, we could not rule it out completely. We sought to address this possibility further by more specifically inhibiting S6K and/or eIF4E. To achieve targeted inhibition of S6 and 4E-BP, as opposed to broader inhibition of mTORC1, we replaced rapamycin in CR culture with the S6K inhibitor PF-4708671, the eIF4E inhibitor 4EGI-1, or both (**Fig 3A**). If rapamycin supports HSC maintenance by inhibiting translation, then an S6K or eIF4E inhibitor should substitute for rapamycin. PF-4708671 effectively inhibits S6K, as measured by reduced S6 phosphorylation in phospho-flow cytometry (**Fig 3B, S2C Fig**), while 4EGI-1 reduces the rate of bulk protein synthesis (**Fig 3C, S2D Fig**). However, it should be noted that the dose of 4EGI-1 that achieves reduced translation also approaches the limit of toxicity [33].

To test whether inhibition of either of these pathways downstream of mTORC1 is sufficient to maintain HSC function, we sorted LSK HSPCs and cultured for 7 d in CHIR99021 plus PF-4708671, 4EGI-1, or both, followed by competitive transplant. While CR-cultured cells engrafted, we detected no chimerism in recipients of any cells cultured without rapamycin (**Fig 3D and 3E**). We conclude that combined inhibition of S6K and eIF4E downstream of mTORC1 is not sufficient to maintain HSC function. Taken together with our findings that CR increases expression of ribosomal protein genes and the rate of global translation, these data argue against inhibition of protein synthesis as a mechanism for maintaining HSCs in cytokine-free culture.

### Mitochondrial mass and activity in maintained HSCs

**Fig 4:**
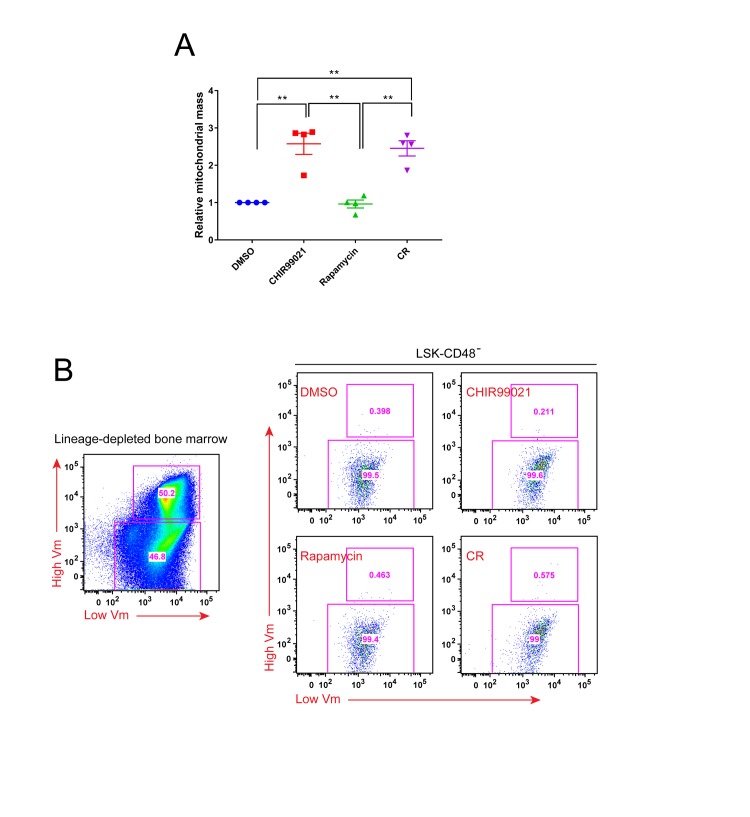
Mitochondrial mass and activity in maintained HSCs. (A, B) Flow cytometric analysis of mitochondrial mass (median fluorescence intensity of Mitotracker Green) relative to DMSO (A) and mitochondrial membrane potential (JC-1) (B) in the LSK-CD48^-^ fraction of lineage-depleted bone marrow cells following 16 h culture. Flow cytometric data shown are representative results of 3 experiments. Error bars represent S.E.M. Vm, mitochondrial membrane potential. Statistical significance was assessed using a one-way ANOVA followed by Tukey’s test for multiple comparisons. * p < 0.05, ** p < 0.005.

In addition to translation, mTORC1 is a known regulator of mitochondria. While mTORC1-driven translation contributes to mitochondrial biogenesis [14], mTORC1 also regulates mitochondrial membrane potential and oxidative capacity independently of translation [15, 34]. As low mitochondrial mass and membrane potential have been correlated with HSC function [17, 35], we explored how GSK-3 and mTORC1 inhibition might affect mitochondria in HSC maintenance. We first measured total mitochondrial mass by MitoTracker Green in the LSK-CD48^-^ fraction of lineage-depleted bone marrow cells after 16 h culture. Cells treated with CHIR99021 either alone or in combination with rapamycin exhibit an increase in mitochondrial mass (**Fig 4A**), consistent with the CHIR99021-mediated increase in translation (**Fig 2G**, **S2B Fig**). However, while mitochondrial mass is generally low in HSCs compared to non-self-renewing progenitors [17, 35], the increase in CR-maintained HSCs argues against a role for mitochondrial mass in the maintenance of HSCs cultured in CR.

In addition to increased mitochondrial mass, increased mitochondrial membrane potential correlates with loss of long-term repopulating capacity of HSCs [16]. We therefore assessed mitochondrial membrane potential, which could allow for the observed maintenance of stemness despite elevated mitochondrial mass in CR-cultured HSCs. Following 16 h culture, we measured mitochondrial membrane potential in LSK-CD48^-^ cells using JC-1, a positively charged dye that accumulates in the negatively charged matrix of polarized mitochondria in a potential-dependent manner [36]. While heterogeneous lineage-depleted bone marrow cells exhibit a distribution between populations of high and low membrane potential, LSK-CD48^-^ cells in all culture conditions are overwhelmingly in the low membrane potential fraction (comparing all culture conditions by one-way ANOVA, p = 0.42) (**Fig 4B**). Thus CR culture increases mitochondrial mass but maintains low mitochondrial membrane potential, compatible with the maintenance of HSC function.

### Reduced cell volume and RNA content in cultured HSCs

mTORC1 integrates nutrient availability and mitogenic stimuli to direct activity of biosynthetic and catabolic pathways, and is thus a major regulator of cell size [37]. Our culture conditions lack serum or exogenous cytokines, known activators of mTORC1, and maintenance of HSCs depends on addition of the mTORC1 inhibitor rapamycin. We therefore assessed cell size following culture of purified HSCs in vehicle, CHIR99021, rapamycin, or CR. By 3 d in culture, all cells were visibly smaller than freshly isolated HSCs (**S3A Fig**). We measured cell area on each day of culture using a micrometer and ImageJ software. Mean cell volume by day 3 decreased 60% in control, rapamycin, and CR-cultured cells and 45% in CHIR99021-treated cells compared to day 0 (**S3B Fig**).

Ribosome biogenesis and protein synthesis are among the most energy-consuming cellular processes. These pathways must therefore be tightly regulated based upon nutrient availability. As cell size generally correlates with ribosome biogenesis [38], we measured total RNA content in HSCs by nanofluidic electrophoresis before and after 3 d culture. RNA was readily detected in uncultured HSCs, but undetectable in an equal number of cultured cells (**S3C Fig**). The low RNA content was confirmed by flow cytometry. We cultured lineage-depleted bone marrow cells in vehicle, CHIR99021, rapamycin, or both. After 3 d, we performed flow cytometric analysis for DNA content by Hoechst 33342 and for RNA content by pyronin Y in the LSK fraction. While freshly isolated HSPCs display a clear distribution through the cell cycle, cells cultured under any condition are overwhelmingly in G0 with markedly reduced total RNA content (**S3D Fig**). We therefore conclude that GSK-3 and mTORC1 inhibition maintains HSC function in cells that have entered a quiescent state.

### Autophagy as a molecular mark of HSC maintenance

**Fig 5:**
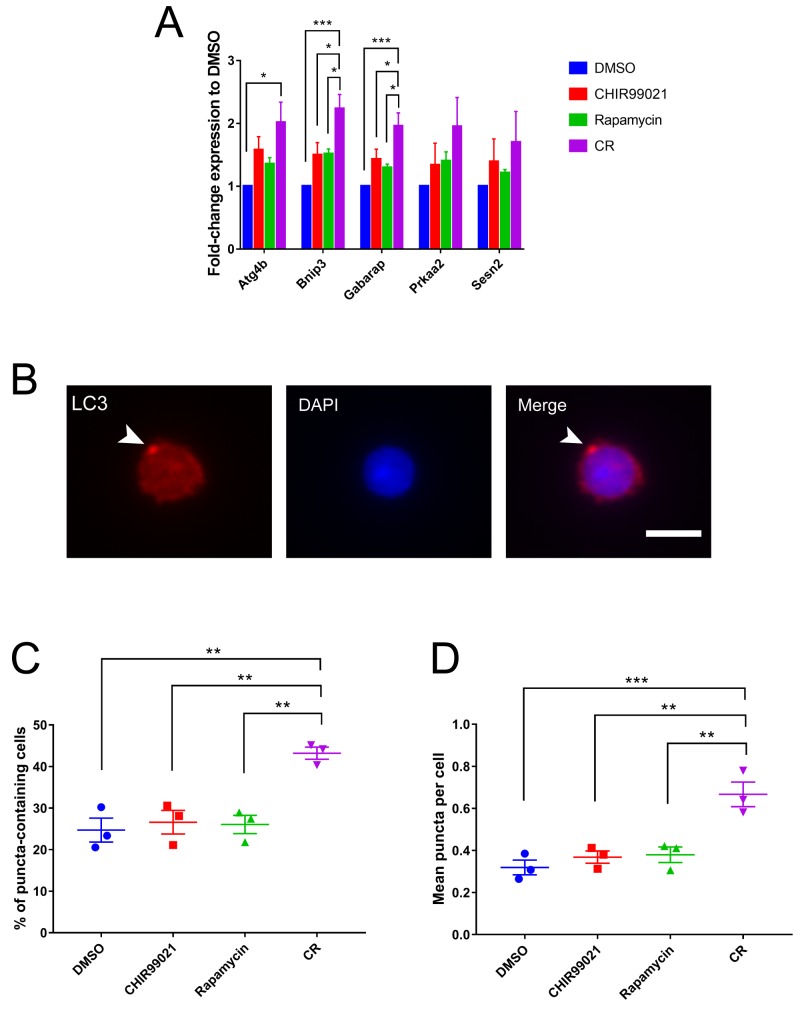
Autophagy as a molecular mark of HSC maintenance. (A) RT-PCR for a pro-autophagic gene signature relative to DMSO in c-Kit^+^ cells following 24 h culture. Data shown are average of 4 experiments. (B-D) HSCs were cultured for 24 h and then fixed for immunofluorescence analysis of LC3 staining. Sample image of LC3-punctum-positive cell at 400× magnification (B). White arrowhead indicates LC3 punctum. Scale bar, 10 μm. Quantification of percentage of LC3-puncta-positive cells (C) and of average number of LC3 puncta per cell (D). For immunofluorescence analyses, n = 85–150 cells counted per treatment group per experimental replicate. Data shown are average of 3 experiments, where each symbol represents the average from one replicate. Error bars represent S.E.M. Statistical significance was assessed using a one-way ANOVA followed by Dunnett’s test for multiple comparisons. ** p < 0.005, *** p < 0.0005.

Quiescent HSCs require autophagy to maintain their capacity for self-renewal; loss of *Atg7* stimulates proliferation of hematopoietic progenitors with a parallel loss of long-term HSCs, leading to a marked myeloproliferative state *in vivo* [24]. Furthermore, suppression of autophagy is a major downstream function of mTORC1, and inhibition of mTORC1 with rapamycin activates autophagy, although typically at concentrations 100 times higher than used here. To examine whether activation of autophagy is specifically associated with maintenance of functional HSCs in culture (e.g., cultured in CR), we performed RT-PCR for a pro-autophagic gene expression signature [27] in c-Kit^+^ cells cultured for 24 h in DMSO, CHIR99021, rapamycin, or CR. Compared to cells cultured under control conditions, cells cultured in CR express significantly higher levels of multiple markers of autophagy, including *Atg4b*, *Bnip3*, and *Gabarap*, with *Prkaa2* and *Sesn2* exhibiting a trend toward higher expression in CR (**Fig 5A**). Notably, expression of these markers is higher in CR than in cells treated with rapamycin alone. This gene signature is consistent with unique activation of autophagy under conditions (simultaneous inhibition of GSK-3 and mTORC1) that maintain functional HSCs in culture.

To confirm activation of autophagy in CR-cultured HSCs, we examined the formation of autophagosomes. As a transition from diffuse to punctate staining for LC3 is a hallmark of autophagosome assembly and activation of autophagy [20, 39], we performed immunofluorescence staining for LC3 puncta in HSCs cultured for 16 h (**Fig 5B**). CR-maintained HSCs exhibited significantly more LC3-puncta-positive cells (**Fig 5C**) and on average more LC3 puncta per cell (**Fig 5D**) compared to vehicle-, CHIR99021-, or rapamycin-treated controls. Taken together with the pro-autophagic gene signature, these results demonstrate that activated autophagy is uniquely enhanced in conditions that maintain HSCs.

## Discussion

mTORC1 regulates cell metabolism and proliferation through multiple pathways. Extensive evidence suggests that mTORC1 activation promotes HSC lineage commitment at the expense of self-renewal [6, 7, 40, 41], but the downstream pathways mediating this effect remain undefined. We have investigated multiple downstream mTORC1 targets to assess which pathways are uniquely activated or inhibited when the GSK-3 and mTORC1 pathways are regulated to maintain HSCs. These studies reveal that autophagy strongly and uniquely correlates with conditions that maintain HSC function *ex vivo*.

We identified a pro-autophagic phenotype by a gene expression signature and by the appearance of LC3^+^ autophagosomes. While mTORC1 is a well-characterized inhibitor of autophagy, the dose of rapamycin used for the studies described here (5 nM) is significantly below the 100–500 nM reported to induce autophagy. Consistently, we only observe a significant increase in autophagy-related gene expression in the presence of rapamycin and CHIR99021 (**Fig 5**). GSK-3 is moreover an established negative regulator of mTORC1 [30], and GSK-3 inhibition might therefore be predicted to block autophagy. However, several compelling lines of evidence suggest a potential mechanism for synergy between the combined inhibition of GSK-3 and mTORC1 to activate autophagy. Recent work reports that GSK-3 inhibits lysosomal acidification independently of mTORC1, blocking autophagy [42]. GSK-3 additionally inhibits autophagy independently of mTORC1 via phosphorylation and cytosolic sequestration of TFEB, the master transcription factor of lysosomal biogenesis [43, 44]. Intriguingly, mTORC1 also inhibits autophagy in part through blocking nuclear translocation of TFEB [45, 46]. It is therefore possible that combined inhibition of mTORC1 and GSK-3 allows TFEB to translocate to the nucleus and activate autophagy.

These findings additionally build on a growing body of literature supporting an essential role for autophagy in the maintenance, activation, and differentiation of multiple stem cell populations, including embryonic [18], epidermal [19], neural [23], skeletal muscle [20, 21], and hematopoietic [24, 25, 27] stem cells. The current speculative model in the field is that autophagy is a vital mechanism of protein and organelle quality control that maintains low levels of reactive oxygen species and protects stem cells from oxidative stress and DNA damage over the lifetime of the organism [47, 48]. As a primary purpose of autophagy is to degrade cytoplasmic contents and generate an internal nutrient pool to sustain protein synthesis during periods of nitrogen starvation [49], autophagic recycling may also serve as an important source of amino acids for stem cells residing in a low-perfusion, reduced-nutrient niche. On the other hand, autophagy can promote stem cell activation and differentiation by degrading multipotency factors and providing biosynthetic materials to support the growth and proliferation required for tissue regeneration [18, 20, 22]. Stem cell fate decisions may thus be directed in part by the extent or targets of autophagic degradation.

In contrast, our results do not support a role for suppression of translation as a mechanism to maintain HSC function *ex vivo*. HSCs maintained in CR display increased ribosomal protein gene expression and an elevated rate of protein synthesis compared to HSCs cultured in control medium or to freshly isolated HSCs (**Fig 2G**, **S2B Fig**). S6K and eIF4E inhibitors correspondingly reduce S6 phosphorylation and the rate of translation but fail to recapitulate *ex vivo* HSC maintenance when combined with GSK-3 inhibition (**Fig 3**). HSCs *in vivo* require a tightly controlled rate of protein synthesis that is regulated in part by the translation initiation inhibitor 4E-BP [12, 13]. The increase in bulk translation that we observe in our cytokine-free, *ex vivo* HSC maintenance system may therefore represent a homeostatic adjustment when HSCs are deprived of signals from their normal microenvironment. It also remains possible that restricting translation may be important to maintain long-term function when HSCs are stimulated to undergo self-renewing cell divisions. However, taken together, our findings of increased global translation in CR medium argue against a role for suppression of protein synthesis as a mechanism for maintenance of HSCs in these *ex vivo* conditions.

We additionally observe a GSK-3-specific increase in mitochondrial mass in cultured HSCs (**Fig 4A**). Like the elevated rate of translation, this is a surprising result given that increasing mitochondrial mass has been correlated with loss of self-renewing function in HSCs [17]. However, mitochondrial membrane potential similarly correlates with HSC function [17], and we find that this is maintained at low levels in functional HSCs *ex vivo* (**Fig 4B**) despite the increase in mitochondrial mass. Our results therefore suggest that preserving the low mitochondrial mass observed in uncultured HSCs is not required to maintain HSC function in CR culture.

All tested culture conditions cause a dramatic reduction in total RNA content and cell size (**S3 Fig**), suggesting a global metabolic shutdown in HSCs upon removal from the niche. At the same time, GSK-3 inhibition (alone or in combination with rapamycin) induces an increase in total protein synthesis (**Fig 2G**, **S2B Fig**), consistent with mTORC1 activation, whereas combined GSK-3 and mTORC1 inhibition uniquely activates autophagy. We speculate that autophagy may provide the biosynthetic material required to support the elevated rate of translation induced by CHIR99021. In this model, only cells that achieve the appropriate balance between anabolism and catabolism resolve these antagonistic metabolic cues and thereby retain self-renewing and multilineage repopulating capacity.

Finally, our studies reveal a signaling network that is sufficient to maintain HSCs cell-autonomously. Extensive crosstalk occurs between HSCs and the microenvironment *in vivo* to contribute to regulation of HSC activity. That progenitors are dispensable in this model system indicates that cell-intrinsic regulation of GSK-3 and mTORC1 is sufficient to maintain HSC function. This finding moreover highlights the potential value of this culture system in characterizing the signaling network that regulates HSC fate decisions, as this analysis would not have been possible to perform on HSCs *in situ*. How HSCs respond to the altered environment outside of the niche is an important consideration as researchers seek methods to expand HSCs to improve the efficacy of HSC transplants.

Our investigation assessed the responses of multiple mTORC1 targets in the context of a signaling network that maintains HSCs. We identified autophagy as a key molecular marker of this signaling network. Future studies may reveal specific targets of these pathways in the maintenance of HSC function.

## Methods and materials

### Mice

C57BL/6 wild-type (CD45.2^+^) and SJL (CD45.1^+^) congenic mice were obtained from the Jackson Laboratory. Mice were bred in-house in a pathogen-free mouse facility at the University of Pennsylvania. Transplant recipients were 8- to 10-week-old CD45.2^+^ females. Donor mice were 6- to 8-week-old CD45.1^+^ males. Animal experiments were performed in accordance with guidelines approved by the Institutional Animal Care and Use Committee (IACUC) at the University of Pennsylvania (IACUC protocol #803107).

### Flow cytometric sorting and analysis of HSCs

Bone marrow cells were flushed from the tibias and femurs of mice with PBS without calcium or magnesium. For detection of LSK cells, whole bone marrow cells were incubated with biotin-conjugated monoclonal antibodies to the following lineage markers: B220 (6B2), CD4 (GK1.5), CD8 (53–6.7), Gr1 (8C5), Mac1 (M1/70), Ter119 (Ter119), and interleukin-7 receptor (IL-7R) (A7R34); FITC-conjugated antibody to Sca1 (Ly6A/E; D7) and APC-conjugated antibody to c-Kit (ACK2). CD48 and CD150 were measured with PE-conjugated antibody to CD48 (HM48-1) and PE Cy7-conjugated antibody to CD150 (TC15-12F 12.2). All antibodies were purchased from eBioscience except for antibody CD150, which was purchased from Biolegend. Biotin-conjugated lineage marker antibodies were detected using streptavidin-conjugated PE Texas Red (ThermoFisher). Antibodies to B220, CD4, CD8, Gr1, Mac1 and IL-7R were diluted 1:550. Antibodies to Ter119 and Gr1 were diluted 1:275. Antibodies to Sca1, c-Kit, CD48, and CD150 were diluted 1:80. Non-viable cells were excluded using the viability dye DAPI (1 μg ml^−1^). Cells were sorted with a FACSAria (Becton Dickinson) automated cell sorter. Analyses were performed on a FACSCanto or LSR II flow cytometer (Becton Dickinson). Data were analyzed using FlowJo software (Tree Star).

### Cytokine-free HSC and HSPC culture

HSCs and HSPCs were cultured as described previously [28]. Briefly, mouse bone marrow cells were harvested and distributed into a single-cell suspension by gently drawing through a 22-gauge needle. Red blood cells were lysed in ammonium chloride–potassium (ACK) buffer. Lineage-depleted bone marrow cells were isolated by incubating whole bone marrow with biotin-conjugated lineage marker antibodies indicated above followed by incubation with Dynabeads M-280 Streptavidin (ThermoFisher) and separation with the EasySep magnet (Stem Cell Technologies). c-Kit^+^ cells were purified with the MACS cell separation kit (Miltenyi Biotec). Isolated HSCs and HSPCs were cultured in X-VIVO 15 (BioWhittaker) supplemented with 1% penicillin and streptomycin (Sigma). CHIR99021 and rapamycin (Cayman Chemical) reconstituted in DMSO were added to final concentrations of 3 μM (CHIR99021) and 5 nM (rapamycin) for all experiments unless otherwise indicated. For inhibition of S6K and eIF4E, cells were cultured in 3 μM CHIR99021 combined with 10 μM PF-4708671 (Tocris Bioscience) reconstituted in ethanol, 50 μM 4EGI-1 (Tocris Bioscience) reconstituted in DMSO, or both. Sorted cells were distributed into 96-well plates with 100 μl medium. One-half volume of medium and full drug volume were replaced every other day.

### Long-term competitive repopulation assays

C57BL/6 (CD45.2^+^) recipient mice were lethally irradiated with a cesium-137 irradiator in two equal doses of 500 rads separated by at least 2 h. The output after culture of 200 SJL (CD45.1^+^) donor cells was mixed with 3 × 10^5^ competitor C57BL/6 bone marrow cells and injected into the retro-orbital venous sinus of anesthetized recipients. Transplanted mice were given antibiotic-containing water for 4 weeks following irradiation. Beginning 4 weeks after transplantation and continuing for 24 weeks, blood was collected from the tail veins of recipient mice and analyzed by flow cytometry for the lineage markers Gr1 (8C5), Mac1 (M1/70), B220 (6B2), CD4 (L3T4), and CD8 (Ly-3) (eBioscience) to monitor engraftment.

### RNA content analysis and cell size measurement

Lineage-depleted bone marrow cells were isolated for analysis of RNA content and cell size on day 0 or after 3 d of culture. At the time of analysis, cells were washed with PBS and stained in LIVE/DEAD Fixable Near IR Dead Cell Stain (ThermoFisher) as directed by the manufacturer, washed, and stained for surface markers indicated. For RNA content analysis by flow cytometry, cells were then washed, resuspended in 20 μg ml^-1^ Hoechst 33342 (Cell Signaling Technologies) with 50 μg ml^-1^ verapamil (Calbiochem) in PBS supplemented with 3% fetal bovine serum (FBS; GE Healthcare Life Sciences), vortexed briefly, and incubated covered at 37°C for 45 min. Pyronin Y (Sigma-Aldrich) was added directly to a final concentration of 1 μg ml^-1^ and cells were briefly vortexed and incubated covered at 37°C for an additional 15 min. Cells were washed and immediately analyzed by flow cytometry.

For size measurement, HSCs were sorted and cultured as described above. Each day, cells were imaged on a Nikon Diaphot microscope equipped with a Nikon DS-Fi1 camera and NIS-Elements F imaging software (Nikon). Cell cross-sectional area was measured in ImageJ (NIH) calibrated to a micrometer, and volume was calculated assuming that cells are spherical.

### Assessment of pS6 and p4E-BP1

To measure phosphorylated S6 and 4E-BP by intracellular flow cytometry, lineage-depleted bone marrow cells were treated with DMSO, 30 μM CHIR99021, 500 nM rapamycin, or both at 37°C for times indicated. Cells were fixed and permeabilized as indicated by Cell Signaling Technology. Cells were stained for the surface markers indicated and for pS6 conjugated to Pacific Blue (Ser235/236 [D57.2.2E], rabbit mAb, final concentration 1:50, Cell Signaling Technology) and p4E-BP1 conjugated to PE (Thr37/46 [236B4], rabbit mAb, final concentration 1:50, Cell Signaling Technology), then analyzed by flow cytometry.

### Assessment of rate of translation

Lineage-depleted cells were cultured as described above for 3 or 24 h, and then O-propargyl-puromycin (OP-Puro; Click-iT Plus OPP AlexaFluor 488 kit, Life Technologies) was added (10 μM) to the medium for an additional 30 min. Cells were washed with PBS and stained with LIVE/DEAD Fixable Near IR Dead Cell Stain (ThermoFisher) as directed by the manufacturer. Cells were fixed in 0.5 ml of 1% paraformaldehyde (Electron Microscopy Sciences) in PBS for 15 min covered on ice. Cells were washed in PBS, then permeabilized in 200 μl PBS supplemented with 3% FBS and 0.1% saponin (Sigma) for 5 min at room temperature. The azide-alkyne cycloaddition was performed using the kit as directed by the manufacturer. After the 30-min reaction, cells were washed in PBS supplemented with 3% FBS, then stained for surface markers indicated and analyzed by flow cytometry.

### Mitochondrial analyses

Lineage-depleted cells were cultured as described above for 16 h and stained for surface markers indicated. Mitochondrial mass and membrane potential were then measured using MitoTracker Green FM (20 nM; Life Technologies) and MitoProbe JC-1 Assay Kit for Flow Cytometry (0.2 μM in X-VIVO 15; Life Technologies), respectively, as indicated by the manufacturer and analyzed by flow cytometry.

### Immunofluorescence microscopy

LSK-SLAM cells were sorted as described above. Cells were cultured for 24 h as described above, then fixed in 4% paraformaldehyde in PBS on glass slides for 30 min at room temperature. Cells were washed twice in PBS, then permeabilized in 0.5% Triton X-100 (ThermoFisher) in PBS for 10 min at room temperature. Cells were then washed twice in PBS and blocked in 10% bovine serum albumin (BSA; Sigma-Aldrich) in PBS. Cells were stained with rabbit anti-LC3 (1:500; MBL) in 5% BSA in PBS supplemented with 0.1% TWEEN 20 (Sigma-Aldrich) for 1 h at room temperature and then overnight at 4°C. After primary stain, cells were washed four times in PBS with 0.1% TWEEN 20. Secondary stain was donkey anti-rabbit IgG conjugated to Alexa Fluor 555 (1:500; ThermoFisher) in 5% BSA in PBS supplemented with 0.1% TWEEN 20, incubated for 1 h at room temperature and protected from light, followed by four washes in PBS with 0.1% TWEEN 20. Cells were counterstained with DAPI (1μg ml^-1^) in PBS for 5 min at room temperature and then washed twice in PBS. Slides were mounted with ProLong Gold antifade reagent (Invitrogen) and cured at room temperature for at least 24 h. Slides were imaged on an Olympus IX81 fluorescent microscope setup with a Hamamatsu Camera Controller C10600. Images were recorded and analyzed using the MetaMorph for Olympus Advanced software.

### RNA isolation and RT-PCR

Cultured c-Kit^+^ or LSK-SLAM cells were centrifuged, lysed in 350 μl RLT Plus Lysis buffer with Reagent DX (Qiagen) and beta-mercaptoethanol (Sigma-Aldrich), and RNA was isolated using the RNeasy Plus Micro kit (Qiagen) as indicated by the manufacturer. For spike-in confirming successful RNA isolation from cultured cells, 150 ng YFP mRNA was added at the time of lysis. RNA concentration was quantified on a NanoDrop 1000 (ThermoFisher). 50 ng total RNA per sample was used for first-strand cDNA synthesis using SuperScript III reverse transcriptase (Invitrogen) as indicated by the manufacturer. Relative gene expression was quantified on a 7900HT Fast Real-Time PCR System (Applied Biosystems) using Power SYBR Green PCR Master Mix (Applied Biosystems). Expression levels of genes of interest were normalized to *β-actin* expression. RT-PCR primers were designed using NCBI Primer-BLAST, and sequences are available upon request.

### Statistical methods

All data are depicted as mean +/- standard error of the mean (S.E.M.). Statistical significance was assessed by one-way ANOVA, followed by post-hoc Tukey’s test or Dunnett’s test for multiple comparisons as indicated. For pairwise comparisons, significance was assessed by Student’s t-test.

## Supporting information

S1 Checklist ARRIVE guidelines checklist. (PDF)

S1 Fig Cultured HSCs generate multilineage reconstitution in competitive transplant. (A-C) HSCs were cultured for 7 d and then transplanted with competitor cells into lethally irradiated hosts. Chimerism data shown are from peripheral blood collected at 24 weeks post-transplant. Multilineage potential of transplanted cells was determined by flow cytometry for donor-derived (CD45.1^+^) cells in the myeloid (A), B (B), and T (C) cell fractions of the blood. Each symbol represents the result from an individual mouse.
(EPS)

S2 Fig HSPC populations display stepwise changes in rate of protein synthesis. (A) Rate of protein synthesis determined by incorporation of OP-Puro in freshly isolated hematopoietic cell populations relative to Lin^+^ cells. (B) Rate of protein synthesis relative to DMSO in the LSK-CD48^-^ fraction of lineage-depleted bone marrow cells following 3 h culture. (C) Flow cytometric analysis of pS6 in the LSK-CD48^-^ fraction of lineage-depleted bone marrow cells treated with PF-4708671 and/or stem cell factor (SCF) (100 ngml^-1^). (D) Flow cytometric analysis of relative rate of protein synthesis in the LSK-CD48^-^ fraction of lineage-depleted bone marrow cells treated with 4EGI-1. Error bars represent S.E.M. Statistical significance was assessed using a one-way ANOVA followed by Tukey’s test for multiple comparisons. * p < 0.05, ** p < 0.005.
(EPS)

S3 Fig Reduced cell volume and RNA content in cultured HSCs. (A, B) HSC size by phase contrast microscopy of freshly isolated cells and after 3 d culture (A) and corresponding volume relative to day 0 over time (B). n = 40–60 cells counted per treatment group per day; **** indicates p < 1.5 × 10^−18^ for all conditions compared to day 0 (Student’s t-test). Images 400× magnification. Scale bar, 20 μm. (C) RNA was isolated from fresh HSCs and from HSCs following 3 d culture. Total RNA content was measured by nanofluidic analysis with up to 70% recovery of spiked-in RNA. (D) RNA content was measured by flow cytometric analysis in the LSK fraction of lineage-depleted bone marrow cells that were freshly isolated and after 3 d culture. Data shown are representative results of 3–5 experiments. Error bars represent S.E.M.
(EPS)
